# Osteoclast-Derived Extracellular Vesicles: Novel Regulators of Osteoclastogenesis and Osteoclast–Osteoblasts Communication in Bone Remodeling

**DOI:** 10.3389/fphys.2018.00628

**Published:** 2018-05-30

**Authors:** Feng-Lai Yuan, Qian-yuan Wu, Zong-Ning Miao, Ming-Hui Xu, Rui-Sheng Xu, Dong-Lin Jiang, Jun-Xing Ye, Fei-hu Chen, Ming-Dong Zhao, Hao-jue Wang, Xia Li

**Affiliations:** ^1^Department of Orthopaedics and Central Laboratory, The Third Hospital Affiliated to Nantong University, Wuxi, China; ^2^Department of Pediatrics, People’s Hospital of Puyang, Puyang, China; ^3^College of Pharmacy, Anhui Medical University, Hefei, China; ^4^Department of Orthopaedics, Jinshan Hospital, Fudan University, Shanghai, China; ^5^The Department of Obstetrics and Gynecology, Wuxi Xishan People’s Hospital, Jiangsu, China

**Keywords:** extracellular vesicles, osteoclasts, communication, osteoclastogenesis, bone remodeling

## Abstract

Extracellular vesicles (EVs), including exosomes, microvesicles, and apoptotic bodies, play an important role in cellular communication during skeletal growth and homeostasis. Bioactive molecules carried by EVs are transported to neighboring and distant cells to trigger a series of signaling cascades influencing bone homeostasis. The bioactive activities of osteoclast-derived EVs include regulation of osteoclastogenesis and osteoclast–osteoblast communication. As osteoclast-derived EVs have the potential to regulate osteoclasts and osteoblasts, their application in osteoporosis and other bone metabolic disorders is currently under investigation. However, very few reviews of osteoclast-derived EVs in bone remodeling regulation have yet been published. This article aims to review recent advances in this field, summarizing a new regulator of osteoclastogenesis and osteoclast–osteoblast communication mediated by osteoclast-derived EVs. We will analyze the major challenges in the field and potential for the therapeutic application of EVs.

## Introduction

Bone is a dynamic, living tissue continually undergoing modeling and remodeling. Many factors, including hormones, growth factors, physical activity, and drugs, can influence bone homeostasis (Xiao et al., 2016; Delgado-Calle et al., 2017; Li X. et al., 2017). Bone remodeling is accomplished via the precise coordination of the activities of the two specific cells: osteoblasts (which deposit calcium into the bone matrix) and osteoclasts (which resorb bone tissues) (Ramasamy et al., 2014; Yuan et al., 2016). Many bone metabolic disorders, such as Paget’s disease or osteoporosis, can be attributed to the imbalance of bone remodeling (Narducci et al., 2011).

Intercellular communication between osteoblasts and osteoclasts plays an important role in the regulation of bone homeostasis (Cao, 2011; Deng et al., 2015), as maintenance of bone requires careful balancing of resorption and formation processes (You et al., 2013). Maintenance and repair of bone, involves both bone degradation, dismantling of old bone matrix, and its replacement with new bone matrix (Kylmaoja et al., 2016). Osteoclasts are derived from hematopoietic progenitor cells by a process controlled by signaling molecules secreted from osteoblasts. Among them M-CSF and RANKL are two crucial signals promoting osteoclast development and survival, thus inducing bone matrix degradation (Li et al., 2010; Negishi-Koga et al., 2011; Chen et al., 2016). To avoid excessive degradation of bone matrix, osteoblasts also secrete OPG to prevent the binding of RANKL to the RANK receptor on osteoclasts (Pneumaticos et al., 2013). The recent characterization of the RANKL–RANK–OPG axis represents an important advance in understanding bone homeostasis (Walsh and Choi, 2014). Moreover, recent studies suggest that Eph receptors on osteoblasts and their ligands on osteoclasts generates bidirectional anti-osteoclastogenic and pro-osteoblastogenic signals into respective cells and presumably influences transition from bone resorption to bone formation (Zhang et al., 2014).

Some factors, such as M-CSF, RANKL, FAS ligand, complement component 3a, and semaphorin produced by osteoblasts have been demonstrated to influence osteoclastic bone resorption. Besides the well-documented regulatory mechanism of osteoblast–osteoclast communication involved in bone resorption, it is now being challenged by several studies supporting the fact that osteoclasts in turn regulate osteoblastic bone formation either by direct cell–cell contact or indirectly via cytokines, however, precisely how osteoclasts regulate osteoblasts is unclear. Accumulating evidence indicates that through either direct cell–cell communication or indirect cytokine-mediated communication, osteoclasts repress osteoblastic bone formation (Ishii et al., 2009; Tang et al., 2009; Negishi-Koga et al., 2011; Li et al., 2016). So far, a growing body of studies has reported that cell–cell communication via EVs is involved in the immune response (Nair and Salomon, 2018). The first description of EVs contain RNA, including microRNA, demonstrates the ability that a cell has to communicate with neighboring cells or with distant cells (Valadi et al., 2007).

Interestingly, communication between osteoclasts and osteoblasts may occur via small membrane-enclosed vesicular particles termed EVs which can fuse with the nearby cell membranes within circulatory pathways (Huynh et al., 2016; Sun et al., 2016). The various roles of EVs, which are released by various cells into the extracellular space, in intercellular communication are only beginning to be understood (Pitt et al., 2016). EVs are broadly classified into three major types: exosomes, microvesicles, and apoptotic bodies, according to their size and presumed biological pathways (Boulanger et al., 2017). Bone-derived exosomes act in the regulatory processes of differentiation and communication of bone cells. EV-mediated communication has been hypothesized to play an important role in the coordination of bone remodeling (Qin et al., 2016; Schmidt et al., 2016; Wang et al., 2016). Earlier studies suggested that osteoblast-derived EVs regulate osteoclast activity (Deng et al., 2015). Recent studies found that EVs play an important role in bone metabolism and the bone microenvironment (Li Q. et al., 2017). Subsequently, studies increasingly show that osteoclast-derived EVs can affect osteoblast formation and function (Ciardiello et al., 2016; Huynh et al., 2016; Sun et al., 2016). In this review, we will summarize recent findings elucidating the mechanism by which osteoclast-derived EVs participate in osteoclast–osteoblast communication in bone development and maintenance.

## Characterization Of Osteoclast-Derived EVs

**FIGURE 1:**
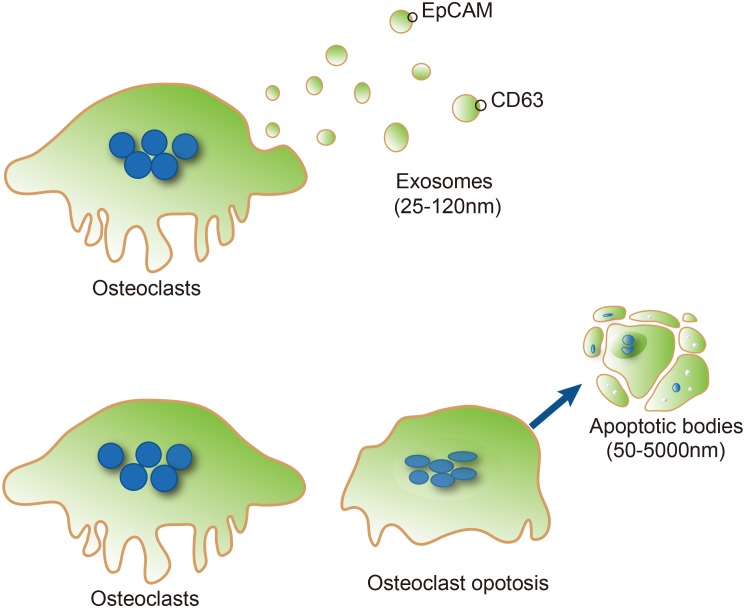
Typical characteristics of osteoclast-derived EVs. Exosomes **(Upper)** are formed from the osteoclast compartment (blue vesicles), and subsequently released into the extracellular space (light yellow vesicles). Epithelial cell adhesion molecule (EpCAM) and CD63 are exosome marker proteins on exosomes. Apoptotic bodies **(Lower)** are usually larger than exosomes (50–5000 nm in diameter) and generated by blebbing of the surface of apoptotic cells from osteoclasts.

So far, two types of osteoclast-derived EVs, exosomes and apoptotic bodies, have been described (Huynh et al., 2016; Li et al., 2016; Sun et al., 2016). Exosomes are defined as small EVs (30–100 nm in diameter) which are released following fusion of endosomes with the plasma membranes (Jørgensen et al., 2013). Transmission electron microscopy revealed that the exosomes released from osteoclasts or their precursors are generally between 25 and 120 nm (mean, 40 nm) in diameter at their widest point (Huynh et al., 2016) and their membranes characteristically include epithelial cell adhesion molecule (EpCAM) and CD63 (**Figure 1**), but not the markers (gp96 calnexin, endoplasmic reticulum) which are common contaminants of EV. Apoptotic bodies are usually larger than exosomes (50–5000 nm in diameter) and are generated by blebbing of the surface of apoptotic cells, resulting in vesicles composed of plasma membranes and organelle with nuclear and cytoplasmic contents (Thompson et al., 2016; **Figure 1**). The apoptosis of osteocyte has been regarded a chemotactic signal to osteoclastic bone resorption. A recent study identified that apoptotic osteocytes are engulfed by osteoclasts during bone resorption, where osteoclasts exhibited TUNEL positive apoptotic bodies share surface markers of the bone matrix, and contain osteoclast vacuoles and osteocytic lacunae.

## The Bioactivity Of Osteoclast-Derived EVs

EVs shed from cells can carry proteins, lipids, mRNAs, and microRNAs (miRNAs) (Penfornis et al., 2016). EVs can transport this cargo into adjacent or distant cells, perhaps altering the behavior of the target cell (Ciardiello et al., 2016; Tkach and Thery, 2016). Huynh et al. (2016) recently reported that EVs released by osteoclasts regulate osteoclastogenesis *in vitro*. In this study, exosome-like EVs from osteoclasts or their precursors were isolated using ExoQuick^TM^ exosome isolation method. The diameter of exosome-like EVs was visualized by transmission electron microscopy. The marker of exosome-like EVs is detectable by Western blot (Huynh et al., 2016). Immunoelectron microscopy revealed RANK on the surface of EVs released from osteoclasts (Huynh et al., 2016). In addition, Western blots indicated that EVs released from osteoclasts contained more RANK than precursors, which could inhibit osteoclastogenesis. On the other hand, ephrinA2 protein was also recently reported to be enriched in osteoclast-derived exosomes (Huynh et al., 2016).

In addition to characteristic proteins, another feature of osteoclast-derived EVs is presence of nucleic acids (such as miRNAs) (Jørgensen et al., 2013; Li et al., 2016; Sun et al., 2016; Xie et al., 2017). miRNAs are a class of small noncoding RNAs of about 22 nucleotides in length (Felekkis et al., 2010). After fusing with the target cellular membrane, internalized miRNAs bind corresponding mRNAs to regulate gene expression (Cong et al., 2017; Maeda et al., 2017). EV-derived miRNAs have been implicated in bone homeostasis (Li et al., 2016), immune responses (Kouwaki et al., 2017), tumor survival (Bell and Taylor, 2017), stress responses (Alexander et al., 2015), and angiogenesis (Wang et al., 2017) by regulating intercellular communication. The transfer of EV-derived miRNAs to a recipient cell where they can affect target gene expression is very important, both in understanding the basic biology of disease progression and for the development of therapeutic approaches. Exosomes released from RANKL-induced RAW 264.7 cells have been demonstrated to play a role in the cross-talk between osteoclasts and osteoblasts (Sun et al., 2016). In that study, microarray analysis identified 13 miRNAs in osteoclast-derived EVs (Sun et al., 2016). Among these miRNAs, miR-148a-3p, miR-183a-5p, miR-214-3p, miR-27a-3p, miR-92a-3p, miR-378a-3p, miR-23a-3p, miR-21a-5p, and miR-16-5p were significantly upregulated, however, miR-155-5p, miR-199a-3p, miR-320-3p, and miR-125a-5p were downregulated in the exosomes released from RANKL-induced osteoclasts. Among the upregulated miRNAs, miR-214-3p has been reported to involved in regulation of osteoclasts and osteoblasts. The miR-214-3P and exosomal-miR-214-3p were further demonstrated to be more abundant in mature mouse osteoclasts than mature osteoblasts. Recent data from abstracts of papers presented in the 40th Annual Meeting of the Canadian Association for Dental Research also showed that miR-146a is abundant in EVs released from osteoclasts.

## Anti-Osteoclastogenic Activity Of Osteoclast-Derived EVs In Osteoclasts

**FIGURE 2:**
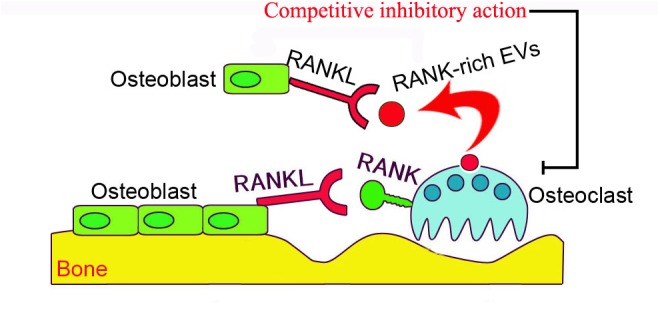
The schematic presentation of osteoclast-derived EV anti-osteoclastogenic activity. RANKL localized on the surface of osteoblasts binds to RANK on the surface of inactive osteoclasts to stimulate osteoclastogenesis. RANK-enriched EVs (exosomes, red vesicles) from inactive osteoclasts may interact with RANKL on osteoblasts to competitively inhibit the association of RANKL and osteoclast-RANK, thus preventing stimulation of the RANK signaling pathway in osteoclasts.

The activity of osteoblast-derived EVs on bone remodeling has been well documented (Xie et al., 2017), however, the mechanism by which osteoclast-derived EVs influence bone remodeling remains uncertain. Huynh et al. (2016) demonstrated that RANKL rich osteoclast-derived EVs inhibit 1,25-dihydroxyvitamin D3 (1,25(OH)2D3)-induced formation of osteoclast-like multinucleated cells in mouse marrow by inhibiting the interaction of RANKL-RANK, and thus the interaction between osteoblasts and osteoclasts. In contrast, incubation of mouse marrow hematopoietic precursor cells directly with RANK rich osteoclast-derived EVs induces osteoclast differentiation, but not generation of TRAP+ multinuclear or giant cells. Thus, RANK rich osteoclast-derived EVs act as inhibitors of osteoclastogenesis through competitively inhibiting the RANK-RANKL interaction in osteoblasts. RANK levels were much higher in osteoclast-derived EVs, and the depletion of EVs containing RANK significantly reduced the inhibition of osteoclastogenesis (**Figure 2**). Furthermore, the depletion of RANK-enriched osteoclast-derived EVs inhibits formation of osteoclast-like cells in 1,25(OH)2D3-treated bone marrow cultures. These data imply that the inhibitory effect of RANK-rich EVs could be applied in bone disease or injury treatment. However, currently we do not know the regulatory activity of RANK-rich EVs from resorbing osteoclasts, which will be of interest for future investigation.

## Communication Between Osteoclasts And Osteoblasts Through Osteoclast-Derived EVs

The maintenance of bone homeostasis relies heavily on cellular communication between osteoblasts and osteoclasts through the RANKL–RANK interaction (Chen et al., 2017). In addition to RANK-RANKL signaling, several other molecules were found to mediate communication between osteoclasts and osteoblasts, and to influence osteoblastic bone formation either directly or indirectly (Matsuo and Irie, 2008). Recent studies revealed that osteoclasts may also communicate with osteoblasts via the fusion of osteoclast-derived exosomes containing miRNAs containing (Ciardiello et al., 2016; Sun et al., 2016). Li et al. (2016) reported that in elderly women with fractures, as in ovariectomized (OVX) mice, miR-214-3p, found in osteoclast-derived exosomes, inhibited osteoblastic bone formation. This activity was tightly associated with elevated levels of serum exosomal miR-214-3p.

**FIGURE 3:**
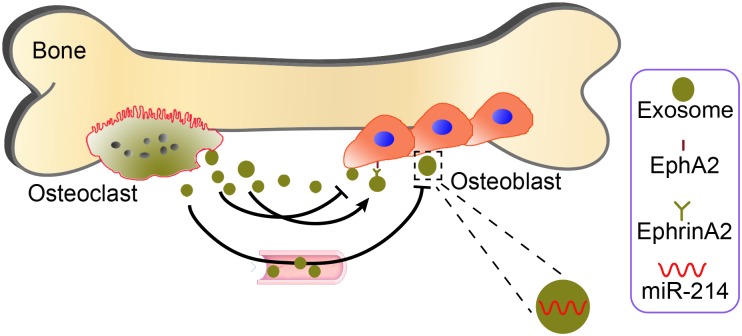
Potential mechanisms by which osteoclast-derived EVs may mediate osteoclast–osteoblast communication. Osteoclast-derived miR-214 packaged into exosomes (yellow green) can inhibit local or distant osteoblast activity via ephrinA2/EphA2 (right aside picture)-mediated osteoclast–osteoblast interactions.

Moreover, compared with wild-type mice, osteoclastic-specific miR-214-3P knock-in (OC-miR-214-3p) mice has lower bone mass and poorly organized trabecular architecture at distal femur. Histomorphometric analysis of the age-related changes and Masson’s trichrome staining also showed less bone formation in OC-miR-214-3p mice than WT mice. In *in vitro* osteoblast/osteoclast co-culture, expression of all the osteoblastic activity-related genes was significantly decreased in OC-miR-214-3p mice than wild type mice. Overexpression of miR-214-3p expression in osteoclasts inhibited osteoblast activity in this co-culture system. These findings suggest that exosomal miR-214-3p could be transferred from osteoclasts to osteoblasts to inhibit osteoblast activity *in vitro*. In a series of tracking experiments, CMV-GFP-CD63 stained osteoclast-derived exosomes were found in co-cultured osteoblasts, indicating that the extracellular osteoclast-derived exosomes effectively reached osteoblasts. *In vivo* injection of purified PKH67 exosomes isolated from the supernatant of OC-miR-214-3p osteoclast dramatically decreased bone formation. In contrast, inhibition of osteoclastic-specific miR-214-3P by osteoclast-targeted antagomiR-214-3p treatment significantly enhanced bone formation (Li et al., 2016). These results indicate that the osteoclast-derived EVs could be important intercellular messengers regulating communication between osteoclasts and osteoblasts to inhibit osteoblastic bone formation. However, this study only suggests a correlation between osteoclast-derived EVs and the intercellular communication, and further investigation of these EVs containing miRNAs in regulating bone remodeling via autocrine and paracrine signaling mechanisms is still needed. Another mechanism was reported by Sun et al. (2016) osteoclast-derived miR-214-containing exosomes could, locally or traveling via the bloodstream to distant sites, inhibit osteoblast activity via ephrinA2/EphA2-mediated osteoclast–osteoblasts interactions. Thus, molecular communication between osteoclasts and osteoblasts are likely to be regulated by osteoclast-derived EVs (**Figure 3**). Although osteoclast-derived exosomes may attach to the membrane of the objective cell to induce intracellular signaling through corresponding receptors, it is recognized as well that osteoclast-derived microvesicles and even osteoclast-derived apoptotic bodies may have largely analogous physiological and pathological roles in intercellular communication. While osteoclast-derived exosomes could be endocytosed via micropinocytosis, phagocytosis, or receptor-/raft-mediated endocytosis, to merge with the membrane of endocytic compartment to emancipate their content into the cytoplasm of the target cells, however, it need to further test *in vitro* or *in vivo*, in addition, the molecular cargo within the osteoclast-derived EVs responsible for this effect is unidentified.

## Perspectives

In summary, bone remodeling is influenced by several hormonal factors. Osteoclast-derived EVs contain multiple key bone-regulatory proteins which can modulate osteoblastic bone formation. This regulative process may reveal a novel mechanism of osteoclast–osteoblast communication. Interestingly, more and more molecules have been isolated from bone-derived EVs, and have been found to influence a number of biological processes through cell–cell communication or via interacting with the extracellular matrix (Deng et al., 2015; Raimondi et al., 2015; Karlsson et al., 2016). The message delivered to target neighboring or more distant cells is determined by the contents of these EVs. The bioactive payload depends on the surrounding microenvironment and the cell of origin during EVs biogenesis (Karlsson et al., 2016). In our opinion, novel osteoclast-derived EVs will continue to be discovered, revealing more and more new molecules that regulate the communication between bone cells. In addition to their role in cell–to–cell communication in normal physiology and disease, recently EVs have been identified as promising biomarkers and novel therapeutic agents in various pathological conditions (Sadovska et al., 2015; Zhang et al., 2016). Compositions of EVs cover most cell-associated biomarkers, including proteins, mRNAs, and miRNAs, which are the focus of EV biomarker research. Simultaneously, EVs have been widely applied in profiling of mRNA, microRNA, and proteins, which may lead to the development of commercial diagnostic kits and potential innovative therapeutic tools(Crossland et al., 2016; Royo et al., 2016). Nevertheless, in order to utilize these osteoclast-derived EVs in the therapy of osteoporosis clinically, further exploration will be required.

## Author Contributions

F-LY, Z-NM, Q-yW, and R-SX conceived the project and designed the experiments. XL and J-XY collected and analyzed the data. All authors developed analytical tools and wrote, edited, and approved the final submission of the manuscript.

## Conflict of Interest Statement

The authors declare that the research was conducted in the absence of any commercial or financial relationships that could be construed as a potential conflict of interest.

## Abbreviations

Eph: erythropoietin-producing human hepatocellular
Ephrins: Eph receptor-interacting proteins
EV: extracellular vesicle
M-CSF: macrophage colony-stimulating factor
NF-κB: receptor activator of nuclear factor kappa B
OPG: osteoprotegerin
RANKL: NF-κB ligand
